# Stem cell therapies for chronic obstructive pulmonary disease: mapping the global clinical trial landscape

**DOI:** 10.1093/stcltm/szag087

**Published:** 2026-11-02

**Authors:** Tingting Xu, Wei Dai, Ziteng Zhu, Yuanrong Dai, Chan Chen, Xiangting Ge

**Affiliations:** Department of Geriatric Medicine, The First Affiliated Hospital of Wenzhou Medical University, Wenzhou 325000, China; Department of Neurorehabilitation, The Second Affiliated Hospital and Yuying Children’s Hospital of Wenzhou Medical University, Wenzhou 325000, China; The Second Clinical Medical College, Wenzhou Medical University, Wenzhou 325000, China; Department of Pulmonary and Critical Care Medicine, The Second Affiliated Hospital and Yuying Children’s Hospital of Wenzhou Medical University, Wenzhou 325000, China; Department of Geriatric Medicine, The First Affiliated Hospital of Wenzhou Medical University, Wenzhou 325000, China; Department of Pulmonary and Critical Care Medicine, The Second Affiliated Hospital and Yuying Children’s Hospital of Wenzhou Medical University, Wenzhou 325000, China

**Keywords:** stem cell therapies, chronic obstructive pulmonary disease, clinical trials, clinical trial landscape, mesenchymal stromal cells

## Abstract

**Background:**

Stem cell therapies have emerged as a promising strategy for repairing structural damage and modulating inflammation in chronic obstructive pulmonary disease (COPD). However, a comprehensive characterization of the global clinical trial landscape remains lacking.

**Methods:**

We retrospectively analyzed clinical trials of stem cell therapy for COPD registered in the Trialtrove database up to October 1, 2025, supplemented by cross-referencing with ClinicalTrials.gov. Key trial characteristics were independently extracted and cross-checked by 2 investigators.

**Results:**

A total of 43 eligible trials were identified. Early-phase studies (Phase I–II) accounted for over 80% of all trials. Mesenchymal stromal cell (MSC)-based therapies and mixed cell populations each accounted for 34.9% of registered trials, followed by bronchial basal progenitor cell-based therapies (23.3%). Among 25 completed trials, only 11 publicly disclosed results. Available findings indicated no major short-term safety concerns for the specific cell products evaluated, whereas efficacy outcomes remained heterogeneous. Academic institutions sponsored most trials, while industry-sponsored studies showed a higher proportion of terminated or inactive trials. The United States and China accounted for the largest numbers of registered trials, although Brazil contributed the greatest number of publicly available outcome reports.

**Conclusions:**

Stem cell therapy for COPD remains in an early exploratory stage, with low result-disclosure rates and limited late-phase evidence. Standardized trial design, improved data transparency, and large-scale randomized controlled trials are urgently needed to advance clinical translation.

## Graphical Abstract

**Graphical Abstract:**
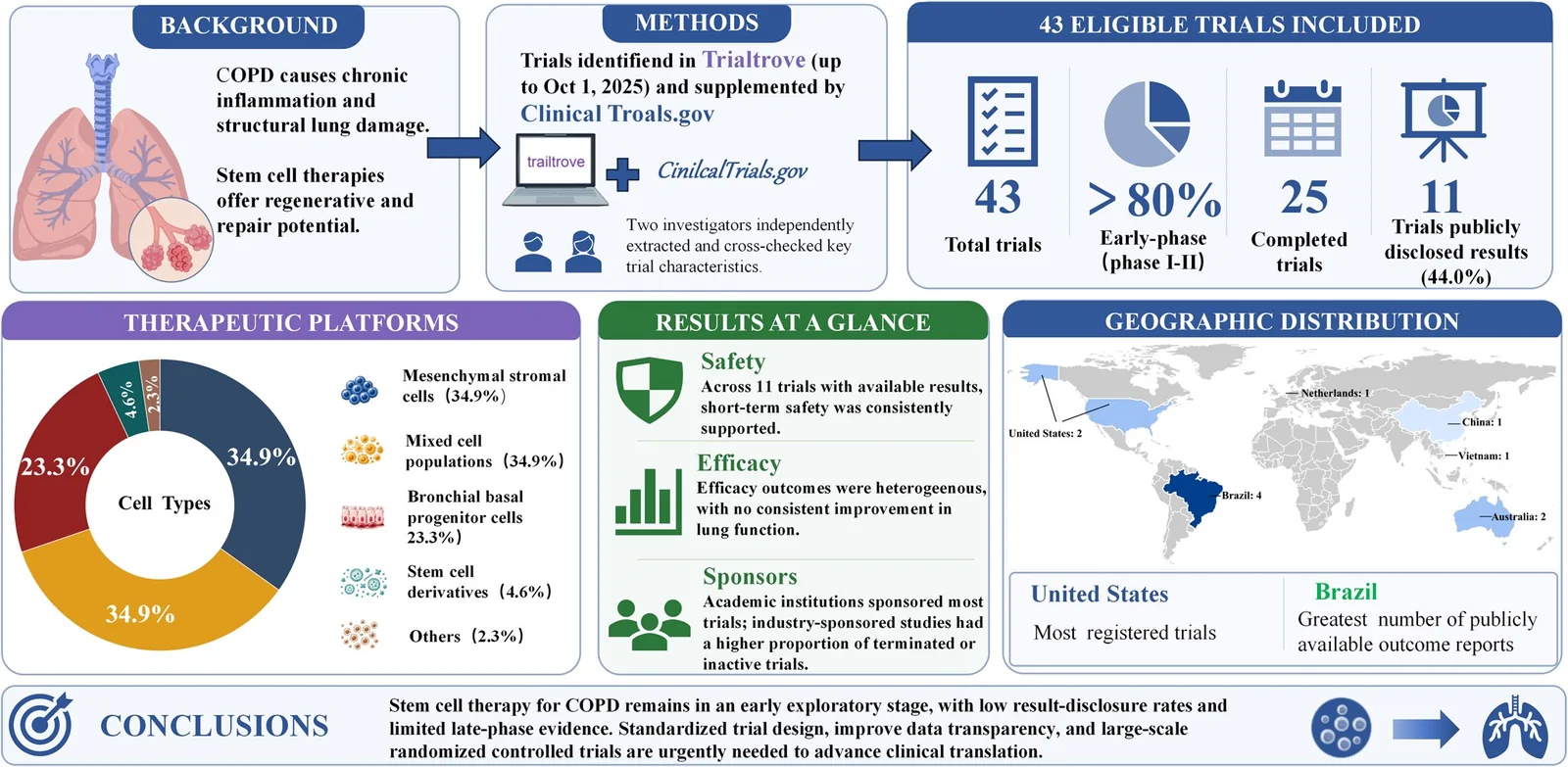

Significance statement Stem cell therapies offer potential regenerative approaches for addressing structural lung damage in chronic obstructive pulmonary disease (COPD). This study maps the global clinical trial landscape of stem cell therapies for COPD, showing that mesenchymal stromal cell-based therapies and mixed cell populations remain the predominant platforms, while bronchial basal progenitor cell-based approaches and cell-free products represent emerging strategies. The current landscape also reveals key barriers to clinical translation, including limited outcome reporting, insufficient advanced-stage studies, and standardization challenges. This analysis may help inform future research and development of regenerative therapies for lung disease.

## Introduction

Chronic obstructive pulmonary disease (COPD), the third leading cause of death worldwide, is characterized by persistent airway inflammation, oxidative stress, and progressive, largely irreversible destruction of alveolar architecture.^1^ Despite advances in pharmacological management, current therapies primarily alleviate symptoms and reduce exacerbation risk but are unable to restore lost alveolar structure or reverse established lung tissue damage.^2^

Stem cell-based therapies have therefore attracted increasing interest as a potential disease-modifying strategy, owing to their immunomodulatory, antioxidative, and regenerative properties.^3^ Preclinical studies have demonstrated that various stem cell populations can attenuate inflammation, modulate immune responses, and improve the pulmonary microenvironment.^4–7^ However, despite these promising biological effects, clinical translation has progressed slowly, and robust evidence supporting durable functional improvement or true structural regeneration in human COPD remains limited.^8^

Stem cell therapies have been investigated in COPD for nearly 2 decades. The first published exploratory clinical study was reported by Ribeiro-Paes et al., who evaluated the safety of autologous bone marrow mononuclear cell (BMMC) infusion in patients with advanced pulmonary emphysema and reported marginal improvements in functional parameters and exercise capacity, although interpretation was limited by the very small sample size.^9^ In contrast, Weiss et al. found no significant benefit of systemic mesenchymal stromal cell (MSC) administration on pulmonary function or quality of life in patients with moderate-to-severe COPD.^10^ Early reviews, including Tzouvelekis et al. and Cheng et al., subsequently synthesized these early clinical and translational findings, highlighting favorable safety signals but persistent challenges in demonstrating consistent spirometric or functional benefit.^11^^,^^12^ Concurrently, preclinical evidence, including the systematic review and meta-analysis by Liu et al., supported the biological rationale for MSC-based investigation in COPD animal models, while also underscoring the need for refined protocols and rigorous clinical validation.^13^ More recently, a comprehensive review has summarized advances in regenerative biology and regenerative therapeutic strategies for COPD, further strengthening the scientific rationale for stem cell-based intervention.^14^ However, substantial heterogeneity exists across clinical trials with respect to cell type, study design, and outcome measures, complicating cross-trial comparison and obscuring the determinants of clinical success or failure.^15^ Consequently, although narrative reviews and mechanistic discussions have provided valuable background, an updated trial-level characterization of the global clinical development landscape remains lacking, particularly with regard to phase distribution, sponsorship structure, geographic activity, and public disclosure of study outcomes.

In this study, we systematically analyzed 43 registered clinical trials investigating stem cell-based therapies for COPD, summarizing trial phases, cell sources, geographic distribution, sponsorship patterns, and result disclosure. By mapping the current clinical landscape, we aim to highlight key challenges and emerging opportunities in pulmonary regenerative medicine for COPD.

## Methods

Clinical trials investigating stem cell-based therapies for COPD were identified using the Trialtrove database (https://pharma.id.informa.com/) through October 1, 2025, and cross-validated using ClinicalTrials.gov (https://clinicaltrial.gov/). Eligible studies evaluated stem cell-based, progenitor cell-based, stromal cell-related, or stem cell-derived therapies for COPD. Interventions were classified into 4 predefined categories: MSC-based therapies, mixed cell populations, bronchial basal progenitor cell-based therapies, and stem cell-derived cell-free products. After exclusion of duplicate and irrelevant records, 43 trials were included. Trial characteristics, including phase, intervention type, trial status, sponsorship, and outcome disclosure, were independently extracted and cross-checked by 2 investigators to ensure data accuracy. Public result disclosure was defined as the availability of trial outcomes through either peer-reviewed publications or registry-reported results. Detailed descriptions of the search strategy, eligibility criteria, study classification, data extraction, and study selection process are provided in the Supplementary Methods. The overall identification and selection process is shown in Figure 1.

**Figure 1.**
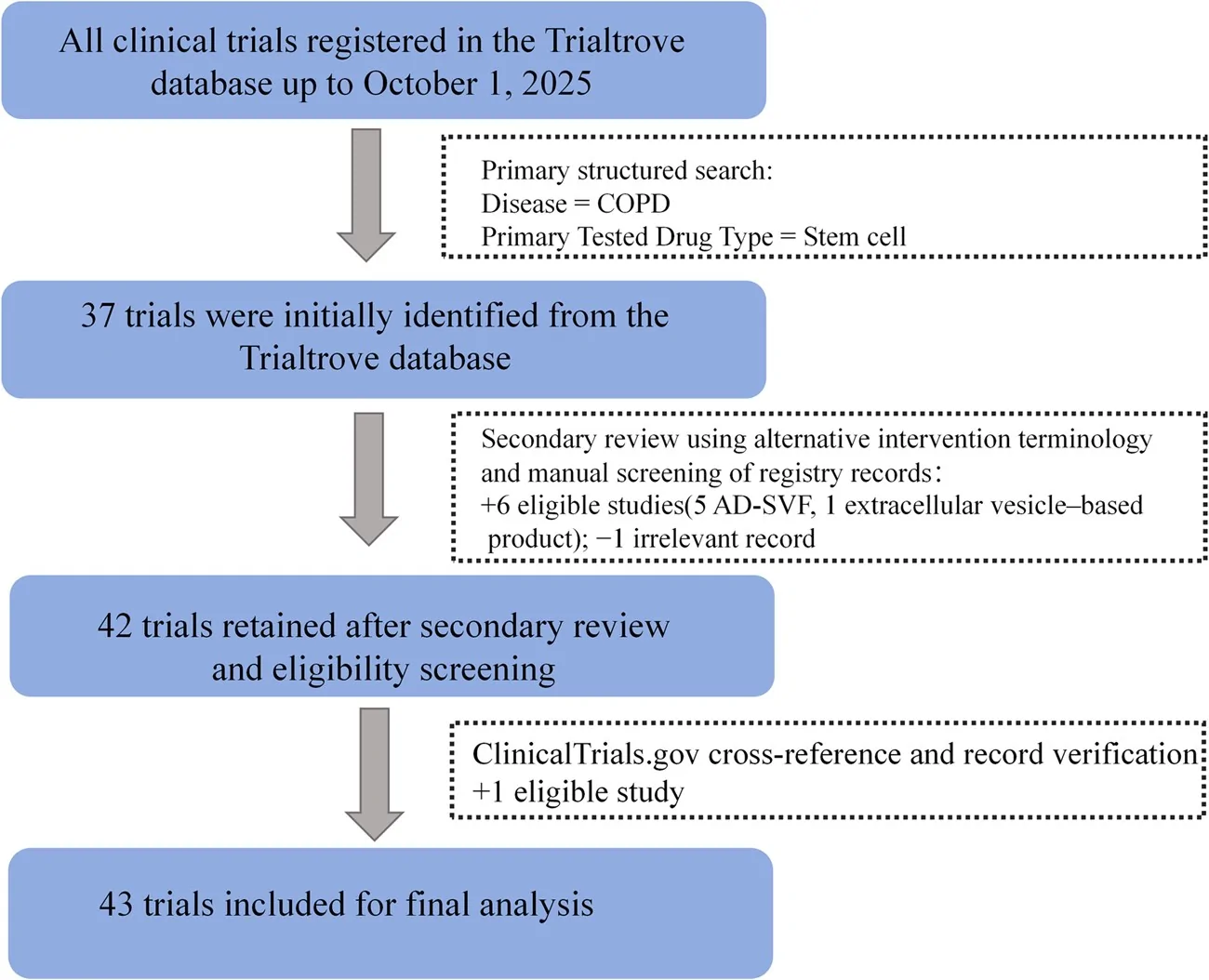
Data processing flow. Registered clinical trials investigating stem cell-based therapies for COPD were identified through a structured Trialtrove search (up to October 1, 2025), followed by a predefined supplementary review consisting of keyword expansion, manual screening of registry records, and cross-referencing with ClinicalTrials.gov. 6 additional eligible studies were identified through the supplementary review, and 1 non-eligible study investigating post-COVID residual pulmonary injury was excluded during manual screening.1 further eligible study was identified through ClinicalTrials.gov cross-referencing, resulting in a final dataset of 43 trials. AD-SVF, adipose-derived stromal vascular fraction.

## Results

### Annual trends and phase distribution

In the registry-based dataset, the earliest eligible clinical trial record was initiated in 2008, whereas the first peer-reviewed clinical report of cell therapy in COPD was published by Ribeiro-Paes et al.^9^ Registered trial activity subsequently increased, reaching a peak in 2017, with secondary peaks observed in 2014 and 2019.

Early-phase studies (Phase I-II) accounted for more than 80% of all trials. Phase III trials began to appear after 2015, while no Phase IV studies have been initiated to date (Figure 2A).

**Figure 2.**
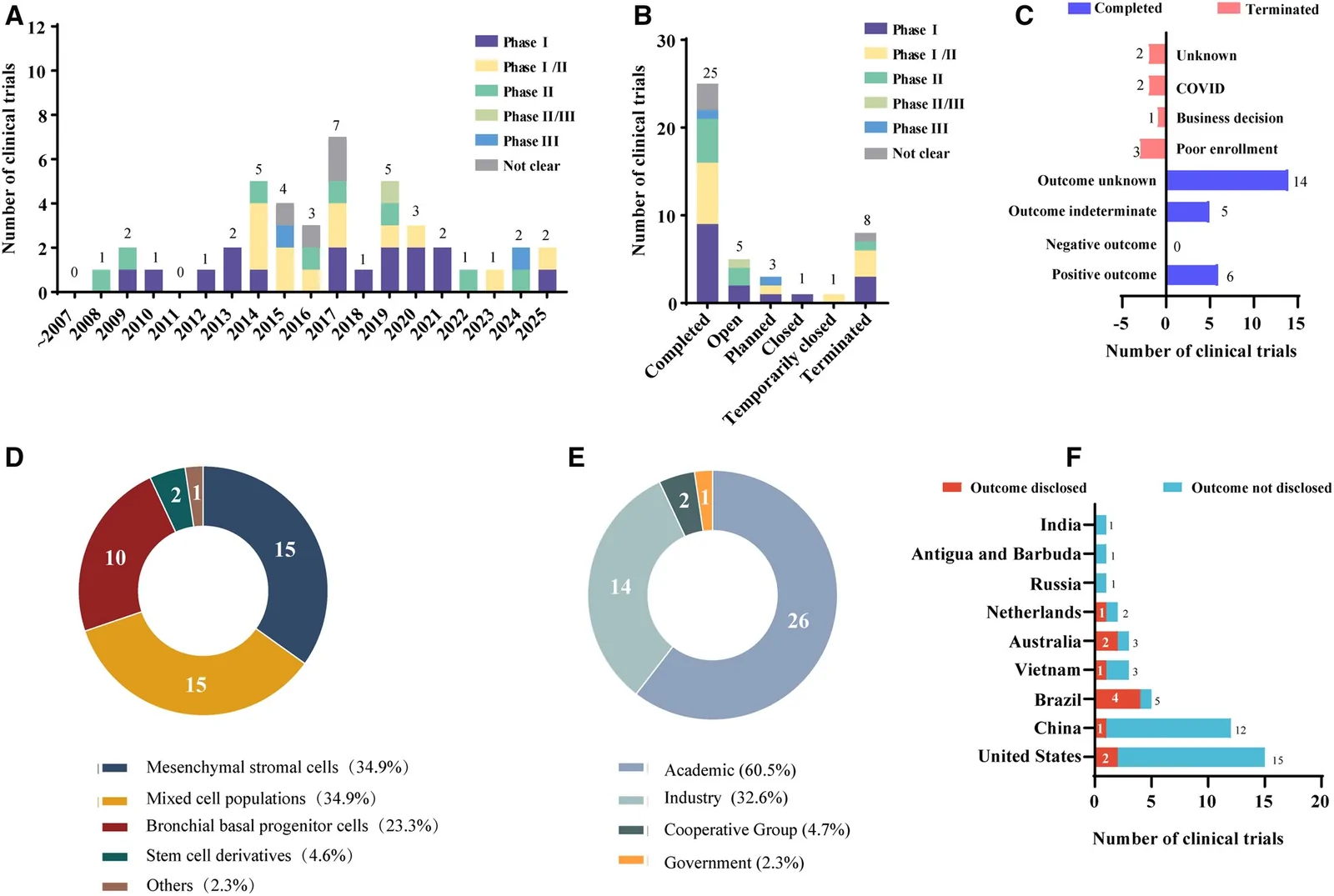
Clinical landscape of stem cell therapy for COPD. (A) Annual distribution of COPD stem cell clinical trials up to October 1, 2025, stratified by study phase. “Not clear” represents studies without explicit phase classification. (B) Overall trial status and phase distribution, including completed, ongoing, planned, closed, temporarily closed, and terminated studies. (C) Outcome disclosure of completed and terminated trials. (D) Distribution of stem cell types used in COPD trials. (E) Types of clinical trial sponsors. (F) Distribution of clinical trial sponsors by headquarters country. Countries are ranked by the total number of registered trials. Orange bars indicate studies with publicly available outcomes, whereas blue bars indicate studies without publicly disclosed results. COPD, chronic obstructive pulmonary disease.

### Trial status and outcome disclosure

Among the 43 identified trials, 58.1% had been completed, 18.6% were open or planned, and 18.6% were terminated prematurely, primarily due to low enrollment (*n* = 3), business decisions (*n* = 1), COVID-19 disruptions (*n* = 2), and unknown reasons (*n* = 2) (Figure 2B and C). Among the 25 completed studies, only 11 had publicly disclosed their results (Table 1). Overall, the publicly available studies reported no major short-term safety concerns for the specific cell products evaluated, whereas efficacy remained heterogeneous. Clinical benefits were mainly reported in symptoms, quality of life, exercise capacity, and DLCO, with limited improvement in spirometric outcomes. Figure S1 summarizes the temporal evolution of publicly available clinical outcome reports in COPD stem cell therapy. The first published clinical study appeared in 2011, followed by a gradual accumulation of publicly available evidence over the subsequent decade. More recent reports demonstrate increasing diversity in therapeutic platforms, including MSC-based therapies, mixed cell populations, bronchial basal progenitor cell-based approaches, and stem cell-derived products.

**Table 1.** Publicly available clinical outcome reports from registered COPD stem cell therapy trials.

Year	Trialtrove ID	Public registry identifier	Evidence source/Ref	Country	Cell/Product type	Phase	Study design	Population	Outcome focus	Key findings
**2011**	126204	NCT01110252	Ribeiro-Paes et al.^9^ (PMID : 21311694)	Brazil	BMMC	II^a^	Single-arm pilot study	Severe COPD	Pulmonary function test	Considering the small sample size of patients (*n* = 4), the results do not support definite conclusions.
**2013**	090634	NCT00683722	Weiss et al.^10^ (PMID : 23172272)	the United States	BM-MSC	II	Randomized, double-blind, placebo-controlled trial	Moderate-to-severe COPD	Adverse events	Safe in moderate-to-severe COPD; no significant efficacy benefit.
**2015**	345623	U1111-1216-4281	Ensaiosclinicos.gov Results database (U1111-1216-4281)	Brazil	Autologous stem cell (BMMC and/or AD-MSC)	III	Randomized-controlled	Moderate-to-severe COPD	Exploratory efficacy	Improved SGRQ, but overall efficacy remained inconclusive.
**2016**	143220	NCT01306513	Stolk et al.^16^ (PMID : 26819296)	the Netherlands	BM-MSC	I	Open-label pilot study	COPD with severe emphysema	Adverse Events	Procedure is safe and feasible; increased pulmonary CD31 expression.
**2017**	188130	NCT01872624	de Oliveira et al.^17^ (PMID : 28186686)	Brazil	BM-MSC	I	Randomized, patient-blinded, placebo-controlled study	Severe COPD	Safety	Combined use of EBV and MSCs appears to be safe in patients with severe COPD.
**2018**	215176	ACTRN12614000731695	Armitage et al.^18^ (PMID : 29348155)	Australia	BM-MSC	I	Open-label pilot study	Mild-to-very severe COPD	Safety and clinical outcomes	Well tolerated, with no attributable adverse effects; no spirometric improvement, but fewer COPD hospitalizations and immunological changes were observed.
**2020**	359842	ISRCTN70443938	Le Thi Bich et a.^19^ (PMID : 32054512)	Vietnam	UC-MSC	II	A pilot clinical trial, without a control group (Single-arm pilot study)	Moderate-to-severe COPD	Safety and quality of life	Improved symptom burden and quality of life; no serious treatment-related adverse events
**2021**	255270	NCT02412332	Squassoni et al.(PMID : 35002228)	Brazil	Autologous stem cell (BMMC and/or AD-MSC)	I/II^a^	Randomized, parallel-group, open-label study.	Moderate-to-severe COPD	Safety and exploratory efficacy	No major safety concerns; efficacy remains inconclusive due to small sample size.
**2021**	295295	NCT03040674	ClinicalTrials.gov results database (NCT03040674)	United States	Autologous stem cells (harvested either from peripheral blood or from bone marrow plus peripheral blood)	Not clear	Observational study	COPD	COPD control questionnaire; Pulmonary function test; Quality of life.	Observational study without a control group; no serious adverse events were reported during follow-up.
**2022**	418271	ACTRN12621001422819	ANZCTR results database (*ACTRN12621001422819*)	Australia	MSC-conditioned medium	I	Open-label, single-group study	Moderately severe COPD	Safety	No adverse events; improved COPD Assessment Test scores and reduced serum CRP
**2024**	303587	NCT03188627	Wang et al.^26^ (PMID : 38354225)	China	Autologous bronchial basal progenitor cell	I/II^a^	Open-label, parallel-group, study	COPD patients who can tolerate bronchoscopy	Safety and DLCO	Improved DLCO and 6-minute walk distance with acceptable safety profile

Year refers to the year in which clinical outcome data became publicly available, either through peer-reviewed publication or registry result disclosure, rather than the year of trial registration. Phase classifications were extracted from trial registry records.

Abbreviations: BMMC, bone marrow mononuclear cells; COPD, chronic obstructive pulmonary disease; CRP, C-reactive protein; DLCO, diffusing capacity of the lungs for carbon monoxide; MSC, mesenchymal stromal cell; UC-MSC, umbilical cord-derived MSC.

^a^Indicate discrepancies between the registry-designated phase and the phase or study-design description reported in the corresponding publication; the registry-designated phase was retained for consistency.

### Stem cell types and sources

A variety of stem cell types were identified across COPD clinical trials (Figure 2D). MSC-based therapies and mixed cell populations were the 2 most frequently investigated platforms, each accounting for 15 of 43 trials (34.9%). Bronchial basal progenitor cell-based therapies represented 23.3% (10/43) of registered studies, followed by stem cell-derived products (4.7%, 2/43) and other cell-based approaches (2.3%, 1/43). Among MSC-based therapies, bone marrow-derived MSCs (BM-MSCs) were the most commonly investigated subtype, whereas adipose-derived stromal vascular fraction (AD-SVF) represented the predominant subtype within the mixed cell population category. Detailed subtype distributions within each platform are summarized in Table S1. Notably, stem cell-derived products have emerged only in recent years, reflecting growing interest in cell-free regenerative strategies.

### Sponsorship and global distribution

Academic institutions sponsored the majority of trials (60.5%), followed by pharmaceutical companies (32.6%), cooperative groups (4.7%), and government agencies (2.3%) (Figure 2E). The United States and China accounted for the largest numbers of registered clinical trials (15 and 12 trials, respectively). However, this pattern was not reflected in the distribution of publicly available outcome reports. Brazil contributed the largest number of publicly available outcome reports (*n* = 4), followed by Australia and the United States (*n* = 2 each), whereas China contributed only 1 publicly available outcome report (Figure 2F).

### Sponsor-stratified trial characteristics

We further performed a sponsor-stratified analysis comparing industry-sponsored and academically sponsored trials (Table 2). Academic-sponsored trials accounted for most completed studies, with 18 of 26 academic trials completed compared with 5 of 14 industry-sponsored trials. In contrast, industry-sponsored trials showed a higher proportion of terminated trials than academic-sponsored trials (28.6% [4/14] vs. 11.5% [3/26]).

**Table 2.** Sponsor-stratified characteristics of chronic obstructive pulmonary disease stem cell therapy trials.

Characteristic	Industry-sponsored trials (*n* = 14)	Academic-sponsored trials (*n* = 26)	Other sponsors (*n* = 3)
**Trial status**			
** Completed**	5 (35.7%)	18 (69.2%)	2 (66.7%)
** Terminated**	4 (28.6%)	3 (11.5%)	1 (33.3%)
** Closed**	1 (7.1%)	0	0
** Planned**	1 (7.1%)	2 (7.7%)	0
** Open**	2 (14.3%)	3 (11.5%)	0
** Temporarily closed**	1 (7.1%)	0	0
**Outcome disclosure among completed trials**			
** Publicly available outcomes**	2/5 (40.0%)	8/18 (44.4%)	1/2 (50.0%)
**Phase distribution**			
** Phase I**	5 (35.7%)	10 (38.5%)	1 (33.3%)
** Phase I/II**	3 (21.4%)	7 (26.9%)	2 (66.7%)
** Phase II**	4 (28.6%)	4 (15.4%)	0
** Phase II/III**	0	1 (3.8%)	0
** Phase III**	1 (7.1%)	1 (3.8%)	0
** Not clear**	1 (7.1%)	3 (11.5%)	0
**Cell/product category**			
** Mesenchymal stromal cells**	5 (35.7%)	8 (30.8%)	2 (66.7%)
** Bronchial basal progenitor cells**	4 (28.6%)	6 (23.1%)	0
** Mixed cell populations**	3 (21.4%)	11 (42.3%)	1 (33.3%)
** Stem cell-derived products**	1 (7.1%)	1 (3.8%)	0
** Others**	1 (7.1%)	0	0

Values are presented as *n* (%) unless otherwise indicated. Percentages were calculated within each sponsor category. Outcome disclosure was defined as the public availability of trial outcomes through either peer-reviewed publications or registry-reported results. Trial phase, status, and cell/product category were extracted from the corresponding trial registry records.

Among completed trials, publicly available outcome information was identified for 2 of 5 industry-sponsored trials and 8 of 18 academic-sponsored trials, corresponding to disclosure rates of 40.0% and 44.4%, respectively. Phase distribution remained dominated by early-stage studies in both sponsor categories. Industry-sponsored trials had a relatively higher proportion of Phase II studies (28.6% vs. 15.4%), whereas academic-sponsored trials were more concentrated in Phase I and Phase I/II studies. Regarding intervention type, mixed cell populations were more common among academic-sponsored trials, whereas MSCs and bronchial basal progenitor cell-based approaches were relatively more frequent among industry-sponsored trials.

## Discussion and conclusion

This study provides a systematic characterization of the global clinical trial landscape for stem cell-based therapies in COPD. Our findings reveal a field that has sustained academic momentum over nearly 2 decades yet remains at an early translational stage, with MSC-based and mixed-cell approaches still predominating and bronchial basal progenitor cell-based or cell-free strategies emerging as exploratory directions.

### Phase distribution and translational barriers

The concentration of trials in Phase I-II reflects a field still primarily engaged in establishing safety and feasibility rather than demonstrating efficacy at scale. Despite a registration peak in 2017, no Phase IV trials have been initiated, and the progression to confirmatory late-phase studies has been markedly slow. This trajectory likely reflects several converging barriers: the inability of current cell-based interventions to consistently improve spirometric parameters such as forced expiratory volume in one second (FEV_1_), which remains the core regulatory endpoint in COPD drug development; substantial inter-trial heterogeneity in cell sourcing, manufacturing, dosing, and delivery routes that complicates cross-study comparison and meta-analytic synthesis; and the absence of validated potency assays or surrogate biomarkers that could serve as reliable predictors of clinical benefit in early-phase settings.

### Safety, efficacy, and the disclosure gap

Across studies with publicly available results, no major short-term safety concerns were reported for the specific cell products evaluated, including MSCs, mixed autologous cell populations, bronchial basal progenitor cells, and stem cell-derived cell-free products. These findings provide preliminary support for the short-term tolerability of the evaluated interventions but should not be generalized to stem cell therapies as a whole. However, the outcome disclosure rate of only 44% among completed trials represents a critical deficiency that undermines the field’s credibility and impedes evidence synthesis. The large proportion of undisclosed completed trials also increases the potential for publication bias, as unpublished studies may include neutral or negative findings that are not reflected in the currently available evidence. Among the publicly available studies, therapeutic benefits were generally modest and were more consistently reflected in symptom improvement than in spirometric measures. However, the current evidence remains limited, and more high-quality clinical data are needed to establish the efficacy of stem cell-based therapies for COPD.

### Sponsorship patterns and reporting transparency

Sponsor-stratified analysis suggests that academic institutions have been the primary drivers of COPD stem cell therapy development. The relatively high industry sponsorship rate (32.6%) combined with premature terminations attributable to business decisions and poor enrollment indicates that pharmaceutical investment in this space, while present, remains fragile and insufficiently sustained. Establishing consensus guidelines for cell product characterization, harmonizing clinical trial endpoints with regulatory expectations, and fostering structured public-private partnerships will be essential to accelerate late-phase development. Outcome disclosure remained incomplete across both sponsor categories, highlighting the need for improved reporting transparency. Mixed cell populations, particularly AD-SVF-based approaches, were more common among academic-sponsored studies, whereas MSC- and bronchial basal progenitor cell-based approaches were similarly represented across sponsor groups. Given the modest subgroup sizes, these findings should be interpreted as descriptive trends rather than definitive sponsor-specific patterns.

### Therapeutic platforms and biological rationale

As illustrated in Figure S2, the major cell-based platforms investigated for COPD differ in their proposed mechanisms of action. MSCs, one of the most extensively studied platforms, are thought to act mainly through paracrine and immunomodulatory effects rather than long-term engraftment or direct tissue replacement. By releasing cytokines, growth factors, and extracellular vesicles, MSCs may reduce inflammation and oxidative stress, regulate immune responses, improve tissue repair.^2^^,^^21^

Mixed cell populations, including BMMCs, stromal vascular fraction, and amniotic- and umbilical cord-derived products, and other heterogeneous autologous cell preparations derived from peripheral blood, bone marrow, or adipose tissue, are heterogeneous in composition. Their potential effects may result from combined paracrine, immunomodulatory, vascular-supportive, and tissue-protective actions, although product consistency and potency may vary.^22^^,^^23^

Cell-free stem cell-derived products, including conditioned media, extracellular vesicle preparations, and other acellular biologics, aim to reproduce the beneficial paracrine effects of regenerative cell therapies without administering viable cells. These products may reduce inflammatory and oxidative injury and offer potential advantages in manufacturing, storage, and standardization.^24^ However, their active components, potency criteria, and optimal dosing remain to be defined.

Bronchial basal progenitor cell therapy represents a more regeneration-oriented strategy. Transplanted cells may promote epithelial repair through engraftment, differentiation, and epithelial replacement,^25^ but may also support endogenous regeneration through paracrine mediators such as EGF, WNT2A, and RSPOs.^26^ A 2024 clinical study provided preliminary evidence that autologous P63^+^bronchial basal progenitor cell transplantation may improve DLCO and 6-minute walking distance without major safety concerns.^26^ Nevertheless, the durability of engraftment, structural regeneration, and long-term clinical benefit requires confirmation in larger randomized trials.

Overall, these mechanistic differences may partly explain the heterogeneity of clinical outcomes across trials.

### Geographic patterns of clinical research

The concentration of trials in the United States and China reflects the advanced regenerative medicine research ecosystems of these countries and their capacity to fund and execute complex cell therapy trials. The participation of Brazil, Vietnam, Australia, and the Netherlands indicates meaningful but limited global engagement. This geographic imbalance warrants attention given that COPD prevalence and mortality are disproportionately high in low- and middle-income countries, where environmental exposures, limited healthcare access, and high smoking rates converge to drive disease burden. Broader geographic participation in future clinical studies may help improve the representativeness of the available evidence and enhance the generalizability of study findings across diverse healthcare settings.

A further finding was that the geographic distribution of publicly available clinical evidence differed from the overall trial registration landscape. Although the United States and China accounted for the largest numbers of registered trials, Brazil contributed the largest number of publicly available outcome reports. These findings highlight the importance of considering both trial registration and publicly available outcomes when assessing the global clinical research landscape of stem cell therapy for COPD.

## Limitations

Several limitations of this analysis should be acknowledged. First, despite supplementation with ClinicalTrials.gov cross-referencing, this registry-based analysis relied primarily on the Trialtrove database and included only studies with identifiable records in the prespecified trial registries. Second, data quality and completeness varied across trials, particularly for older registrations and terminated studies where protocol details were incompletely recorded. Third, this is a descriptive landscape analysis, no formal efficacy or safety meta-analysis was performed, and the small number of trials with disclosed results renders quantitative synthesis neither feasible nor reliable.

## Conclusion and future perspectives

Stem cell therapy for COPD remains an early-stage and heterogeneous field, with MSC-based and mixed-cell therapies representing the predominant clinical platforms. Meanwhile, bronchial basal progenitor cell-based approaches and cell-free biotherapeutics have emerged as promising regenerative strategies, although their long-term clinical efficacy remains to be established. Advancing stem cell-based therapies toward clinical applications will require adequately powered multicenter randomized controlled trials, standardized cell products and regulatory-aligned endpoints, improved result disclosure, and harmonized manufacturing and quality control standards. The favorable safety signals established across nearly 2 decades of early-phase investigation provide a firm foundation from which the field can now advance toward confirmatory efficacy evidence and, ultimately, toward accessible disease-modifying treatments for the hundreds of millions of patients worldwide living with COPD. In parallel, emerging experimental platforms, including 3-dimensional (3D) culture systems and organ-on-a-chip technologies, are expected to provide increasingly physiologically relevant models for investigating COPD pathophysiology, optimizing regenerative strategies, and facilitating translation of stem cell-based therapies into clinical practice.

## Supplementary Material

szag087_Supplementary_Data

## Data Availability

The data supporting this study are provided in the article and its Supplementary Materials. Trial information was primarily obtained from Trialtrove (https://pharma.id.informa.com/), a subscription-based database, and supplemented and cross-validated using publicly accessible trial registries and published literature.
