# Supplementary material for: Stem cell therapies for chronic obstructive pulmonary disease: mapping the global clinical trial landscape
Source: Stem Cells Transl Med. 2026 Nov 2;15(11):szag087. doi: 10.1093/stcltm/szag087 (PMC13726808; doi:10.1093/stcltm/szag087)
Supplement: Supplementary file 1 [file szag087_supplementary_data.docx]

**Supplemental Material**

**Stem Cell Therapies for Chronic Obstructive Pulmonary Disease: Mapping the Global Clinical Trial Landscape**

Tingting Xu^#1^, Wei Dai^#3^, Ziteng Zhu^4^,Yuanrong Dai^2^, Chan Chen*^1^, Xiangting Ge*^2^

1. Department of Geriatric Medicine, The First Affiliated Hospital of Wenzhou Medical University, Wenzhou, 325000, China.
2. Department of Pulmonary and Critical Care Medicine, The Second Affiliated Hospital and Yuying Children's Hospital of Wenzhou Medical University, Wenzhou, 325000, China.
3. Department of Neurorehabilitation, The Second Affiliated Hospital and Yuying Children's Hospital of Wenzhou Medical University, Wenzhou, 325000, China.
4. Wenzhou Medical University, Wenzhou, 325000, China.

*Correspondence to:

Xiangting Ge, PhD, Department of Pulmonary and Critical Care Medicine, the Second Affiliated Hospital and Yuying Children's Hospital of Wenzhou Medical University, No. 109 Xueyuan West Road, Lucheng District, Wenzhou, 325000, China. Email [xiangtingge@163.com](mailto:huangxiaoying@wzhospital.cn)

Chan Chen, Professor, Department of Geriatric Medicine, The First Affiliated Hospital of Wenzhou Medical University, Shangcai Village, Nanbaixiang, Ouhai District, Wenzhou, 325000, China. Email [chenchan99@126.com](mailto:huangxiaoying@wzhospital.cn)

^#^Contributed equally

## Supplementary Methods

## Data source and search strategy

Clinical trials of stem cell-based therapies for COPD were identified using the Trialtrove database (Informa Pharma Intelligence, <https://pharma.id.informa.com/>). The primary search used the filters “Primary Tested Drug Type = Stem Cell” and “Disease = Chronic Obstructive Pulmonary Disease (COPD),” retrieving all records up to October 1, 2025. The Trialtrove database integrates trial registrations from multiple international registries, including ClinicalTrials.gov, the EU Clinical Trials Register, the Chinese Clinical Trial Registry (ChiCTR), the Australian New Zealand Clinical Trials Registry (ANZCTR), and the International Standard Randomised Controlled Trial Number Register (ISRCTN) registry.

## Supplementary search and secondary review

To address heterogeneity in terminology and potential underascertainment, a structured secondary review was performed. This followed a predefined stepwise process: (1) Additional searches within Trialtrove were performed using the following intervention-related terms: “stromal cell,” “stromal vascular fraction,” “progenitor cell,” “conditioned medium,” “exosome,” and “extracellular vesicle”; (2) Manual screening of trial titles, intervention descriptions, and registry records; (3) Cross-validation with ClinicalTrials.gov to confirm eligibility and trial identity.

All additional studies were identified through database keyword expansion and registry-level screening.

## Eligibility criteria and classification

Trials were included if they evaluated stem cell-based, stromal cell-related, progenitor cell-based, or stem cell-derived therapies in COPD. Interventions were classified into four predefined categories: (1) Mesenchymal stromal cell (MSC)-based therapies; (2) Mixed cell populations, including bone marrow mononuclear cells (BMMCs), SVF, amniotic- and umbilical cord-derived products, adult stem cells (from peripheral blood and/or bone marrow and/or **adipose**); (3) Bronchial basal progenitor cell-based therapies; (4) Stem cell-derived cell-free products (conditioned media or extracellular vesicles)

BMMC-based interventions were classified under mixed cell populations due to their heterogeneous mononuclear composition.

## Data extraction and outcome definition

Two investigators independently extracted trial characteristics, including phase, intervention type, sponsor, geography, status, and outcome reporting.

Result disclosure was defined as the public availability of trial outcomes through either peer-reviewed publication or registry-reported results.

## Study selection summary

Initial screening identified 37 trials. After secondary review, six additional trials were identified, one irrelevant record was excluded, and one further study was added via ClinicalTrials.gov cross-validation, yielding a final dataset of 43 trials.

## Supplementary Figure Legend

**Figure S1. Timeline of publicly available clinical outcome reports from COPD stem cell therapy trials.**

The timeline summarizes all publicly available clinical outcome reports identified from completed COPD stem cell therapy trials between 2011 and 2024, including both peer-reviewed publications (solid boxes; n = 8) and registry-disclosed results without corresponding peer-reviewed publications (dashed boxes; n = 3). Each entry indicates the year of publication or registry result disclosure, first author (where applicable), trial registration number, and therapeutic platform. Border colors denote intervention categories: green, mesenchymal MSC-based therapies; blue, mixed cell populations; red, bronchial basal progenitor cell-based therapies; and orange, stem cell-derived products. Key findings summarize reported safety information and, where available, efficacy findings.

Abbreviations: AD-MSC, adipose-derived mesenchymal stromal cells; BMMC, bone marrow mononuclear cells; CAT, COPD Assessment Test; CRP, C-reactive protein; DLCO, diffusing capacity of the lung for carbon monoxide; EBV, endobronchial valve; MSC, mesenchymal stromal cell; PB, peripheral blood; BM, bone marrow; QoL, quality of life; 6MWD, 6-minute walk distance.

**Alt text:** Timeline of publicly available clinical outcome reports from chronic obstructive pulmonary disease stem cell therapy trials between 2011 and 2024, showing publication type, therapeutic platform, and key reported safety and efficacy findings.

**Figure S2. Proposed mechanisms of different stem cell–based therapeutic platforms for COPD.** COPD is characterized by chronic airway inflammation, oxidative stress, and progressive alveolar destruction. MSC-based therapies primarily act through paracrine and immunomodulatory mechanisms to suppress inflammation, regulate immune responses, reduce oxidative stress, and promote tissue repair. Mixed cell populations may exert combined paracrine, immunomodulatory, angiogenic, and tissue-supportive effects. Bronchial basal progenitor cells represent a regeneration-oriented strategy that may promote airway epithelial regeneration, alveolar re-epithelialization, and paracrine-mediated lung repair, although the relative contributions of these mechanisms remain unclear. Stem cell-derived cell-free products aim to reproduce the beneficial paracrine effects of stem cells through delivery of soluble mediators and extracellular vesicles.

Abbreviations: AD-SVF, adipose-derived stromal vascular fraction; BMMC, bone marrow mononuclear cell; BM-MSC, bone marrow-derived mesenchymal stromal cell; MSC, mesenchymal stromal cell; ROS, reactive oxygen species; UC-MSC, umbilical cord-derived mesenchymal stromal cell.

**Alt text:** Schematic showing the proposed mechanisms of four stem cell-based therapeutic platforms for chronic obstructive pulmonary disease. Mesenchymal stromal cells mainly exert paracrine, immunomodulatory, antioxidant, and reparative effects; mixed cell populations may exert combined paracrine, immunomodulatory, angiogenic, and tissue-supportive effects; bronchial basal progenitor cells may promote epithelial regeneration and repair; and stem cell-derived cell-free products aim to mimic the beneficial paracrine effects of stem cells.

**Table S1. Complete list of 43 clinical trials of stem cell-based therapies for COPD (2008–2025）**
